# SG-APSIC1107: Effectiveness of interventions increasing surgical hand hygiene compliance at Hung Vuong Hospital

**DOI:** 10.1017/ash.2023.43

**Published:** 2023-03-16

**Authors:** Anh Dinh, Ngo My Nhung, Tran Thi My Hanh, Ngo Thi Thanh Tham, Bui Thi Thuy Tien

**Affiliations:** Obstetrics and Gynecology, Hung Vuong Hospital, Ho Chi Minh, Vietnam; Hung Vuong Hospital, Ho Chi Minh, Vietnam; Hung Vuong Hospital, Ho Chi Minh, Vietnam; Hung Vuong Hospital, Ho Chi Minh, Vietnam; Hung Vuong Hospital, Ho Chi Minh, Vietnam

## Abstract

**Objectives:** Surgical handwashing is one of the most important measures to prevent surgical site infection (SSI). We evaluated the effectiveness of the intervention program on surgical handwashing compliance of healthcare workers (HCWs) at Hung Vuong Hospital. **Methods:** This research was conducted from July 2019 to November 2019 in 3 phases. In the first phase, we determined the surgical handwashing compliance rate before the intervention. In the second phase, we implemented an intervention bundle as follows. We provided reminders of compliance in the form of video screen and automatic timers at surgical handwashing sinks. We provided links and QR codes for online access and live streaming of instructional videos on implementation of the hospital’s surgical hand sanitation procedures in the surgical handwashing area. We conducted direct monitoring to remind and guide HCWs to follow the procedures in combination with camera surveillance to accurately reflect compliance. Finally, we provided feedback in multiple steps: feedback to individual, feedback to head of department or department heads, cited names in briefings and sent names to the general planning department to suspend surgery privileges. In the third phase, we re-evaluated the surgical handwashing compliance rate after the intervention. **Results:** The total number of surgical handwashing checklists observed before and after the intervention was 787. The surgical handwashing compliance rate improved significantly from 48.8% to 71.8% (PR, 2.7; 95% CI, 1.98–3.57; *P* < .01). The compliance rate in camera monitoring also increased from 22.1% to 57.9% (PR, 4.8; KTC 95%, 3.14–7.47; *P* < .01). The compliances rates of both surgeons and scrub nurses improved significantly after the intervention (*P* < .01). Conducting the new surgical handwashing procedure increased from 90.2% to 99.5% after this intervention. **Conclusions:** This intervention program improved surgical handwashing compliance of HCWs.

